# The solute transport and binding profile of a novel nucleobase cation symporter 2 from the honeybee pathogen *Paenibacillus larvae*

**DOI:** 10.1002/2211-5463.12488

**Published:** 2018-07-23

**Authors:** Amanda J. Stoffer‐Bittner, Candace R. Alexander, Douglas W. Dingman, George S. Mourad, Neil P. Schultes

**Affiliations:** ^1^ Department of Biology Indiana University‐Purdue University Fort Wayne IN USA; ^2^ Department of Entomology The Connecticut Agricultural Experiment Station New Haven CT USA; ^3^ Department of Plant Pathology & Ecology The Connecticut Agricultural Experiment Station New Haven CT USA

**Keywords:** heterologous complementation, nucleobase, nucleobase cation symporter, *Paenibacillus larvae*, PlUacP, transporter

## Abstract

Here, we report that a novel nucleobase cation symporter 2 encoded in the genome of the honeybee bacterial pathogen *Paenibacillus larvae* reveals high levels of amino acid sequence similarity to the *Escherichia coli* and *Bacillus subtilis* uric acid and xanthine transporters. This transporter is named *P. larvae* uric acid permease‐like protein (PlUacP). Even though PlUacP displays overall amino acid sequence similarities, has common secondary structures, and shares functional motifs and functionally important amino acids with *E. coli* xanthine and uric acid transporters, these commonalities are insufficient to assign transport function to PlUacP. The solute transport and binding profile of PlUacP was determined by radiolabeled uptake experiments via heterologous expression in nucleobase transporter‐deficient *Saccharomyces cerevisiae* strains. PlUacP transports the purines adenine and guanine and the pyrimidine uracil. Hypoxanthine, xanthine, and cytosine are not transported by PlUacP, but, along with uric acid, bind in a competitive manner. PlUacP has strong affinity for adenine *K*_m_ 7.04 ± 0.18 μm, and as with other bacterial and plant NCS2 proteins, PlUacP function is inhibited by the proton disruptor carbonyl cyanide m‐chlorophenylhydrazone. The solute transport and binding profile identifies PlUacP as a novel nucleobase transporter.

**Abbreviations**
AzgA: aza‐guanine purine transporter A
COG: cluster of orthologous groups
NCS1: nucleobase cation symporter 1
NCS2: nucleobase cation symporter 2
PlUacP: *P. larvae* uric acid permease‐like protein
UapA: uric acid/xanthine transporters A

*Paenibacillus larvae* is a Gram‐positive bacterium that is the causal agent of American foulbrood of *Apis millifera* Linnaeus 1, 2. *P. larvae* causes disease on larvae but not adult bees 3, 4. Larvae ingest *P. larvae* endospores (the infectious agent) from contaminated royal jelly and are most susceptible at twelve to thirty‐six hours after hatching 4. Spores germinate and greatly proliferate in the larval midgut prior to infection across the midgut epithelium 5. Recently, it was shown that spore germination can be triggered by L‐tyrosine and uric acid *in vitro* 6. Uric acid, a waste product from purine degradation and metabolized proteins, accumulates to high levels in the midgut since the mid and hindgut are not yet connected in young larvae 7. Once the midgut epithelium is breeched, bacterial enter and proliferate in the hemocoel, producing extracellular proteases 8 and toxins 9 leading to cell death and forming a ropey mass of cellular degradation. Depletion of food supplies causes the bacteria to produce highly resistant spores. Worker bees sense the decay, open the brood chamber, remove the carcass, and become coated with spores that are available to infect subsequent larvae 3, 4. The abundance of cellular metabolites, including nitrogen‐rich nucleobases, nucleosides, and nucleotides, in the hemoceol of infected larvae offers *P. larvae* ample opportunity to import valuable nutrients for rapid proliferation.

Nucleobase transporters, ubiquitous among plants, fungi, and bacteria, belong to five independent families. The families include nucleobase cation symporter 1 (NCS1), found in bacteria, fungi, and plants; nucleobase cation symporter 2 (NCS2) family, also known as the nucleobase ascorbate transporter (NAT) family, present in bacteria, fungi, and plants; the closely related aza‐guanine‐like transporters (AzgA) found in bacteria, fungi, and plants and plant‐specific purine permease and ureide permease families 10, 11, 12, 13, 14, 15, 16. The NCS2 family has been the most extensively studied 14. In plants, NCS2 tend to have broad and mixed solute transport and binding profiles including combinations of xanthine, uric acid, uracil, adenine, guanine, cytosine, and hypoxanthine [17, 18 & Schultes and Mourad pers comm]. Plant cells have complex nucleobase biochemistry including *de novo* synthesis, salvage, and catabolic pathways that rely upon different intercellular compartments with diverse environments. In contrast, single‐cell microbes primarily use nucleobase transporters for acquisition of these nitrogen‐rich compounds from the external environment. Unlike plants, microbes have NCS2 with very restricted solute transport and binding profiles. The fungus *Aspergillus nidulans* contains two NCS2—UapA and UapC—that transport uric acid and xanthine 14 and the adenine–hypoxanthine–guanine transporter AzgA 19, 20. Both UapA and AzgA have been extensively studied with dozens of mutations characterized at the biochemical level 21, 22. *Escherichia coli* contains ten NCS2 with narrow solute transport profiles: the cluster of orthologous group COG2252 contains EcGHXQ (YgfQ) and EcGHXP (YjcD) that transport guanine and hypoxanthine 23 and EcAdeP (YieG) and EcAdeQ (YicO) that transport adenine 23; the cluster of orthologous group COG2233 contains EcUraA that transports uracil 24, EcXanP(YicE) and EcXan Q (YgfO) that transport xanthine 25, EcUacT that transports uric acid 26, while EcRutG 27 and EcYbbY are of unknown function 28. The three‐dimensional structures of EcUraA with uracil in the inward‐open and occluded states 24, 29 and that of an inward‐facing AnUapA with xanthine 30 have recently been solved. In addition, a number of EcNCS2 proteins have undergone extensive mutagenesis and biochemical characterization 24, 25, 26, 31, 32. With this impressive backdrop, we functionally characterize a novel NCS2 transporter from *Paenibacillus larvae* which we named PlUacP.

## Materials and methods

### Microbial strains and growth conditions

*P. larvae* subspecies *larvae* strain NRRL B‐3650 was grown on MYPGP media 33. *Escherichia coli strain* DH5α (*fhuA2 lacΔU169 phoA glnV44 Φ80’ lacZΔM15 gyrA96 recA1 relA1 endA1 thi‐1 hsdR17*) was grown on LB media with 50 mg·mL^−1^ carbenicillin and used for molecular cloning. *S. cerevisiae* strains RG191 [*MAT*α*, fyc2Δ::kanMX4, his3Δ1, leu2Δ0, met15Δ0, ura3Δ0*] 34 and NC122‐sp6 [*MAT*α *leu2 fur4Δ*] 35 were grown in synthetic complete (SC) medium—leucine (Sigma‐Aldrich, St. Louis, MO) at 30 °C. Yeast transformations were performed by the lithium acetate method 36.

### Nucleic acid manipulations

Genomic DNA was isolated from *P. larvae* strain B‐3650 using QIAamp Tissue kit (Qiagen Inc. Santa Clarita, CA). The coding region for PlUacP was PCR‐amplified from genomic DNA using oligonucleotides *PlXPa* 5’ cccaagcttctcgagatgcgtaaaagcaaagtacttacc 3’ and *PlXPb* 5’ ataagaatgcggccgcttaagcggcatgaacgttttctgc 3’ using conditions 94 °C 2 min; 94 °C 15 s; 55 °C 30 s; 70 °C 2 min; repeat 30X; 72 °C 10 min. The resulting PCR product was purified using QIAquick PCR Purification Kit (Qiagen Inc. Santa Clarita, CA) and, along with yeast expression vector pRG399, cut with restriction endonucleases *Xho* I and *Not* I, ligated and transformed into *E. coli* strain DH5α to generate plasmid pNS515. DNA sequence analysis was performed at W.M. Keck Biotechnology Resource Laboratory at Yale School of Medicine (New Haven CT, USA) to verify sequence integrity. pNS515 was transformed into yeast strains RG191 and NC122‐Sp6 and transformants selected on SD—leucine media. RNA was isolated from *P. larvae* strain B‐3650 culture grown in MYPGP to an OD_600_ of 0.64 using the RNeasy Protection Kit/RNA protect Bacterial Reagent (Qiagen Inc. Santa Clarita, CA, USA) and treated with RNase free DNaseI (Roche, Indianapolis, IN, USA). Genomic DNA, DNaseI‐treated total RNA, and DNaseI‐treated total RNA with Invitrogen SuperScript One‐Step RT‐PCR treatment (ThermoFisher, Waltham, Massachusetts, USA) were amplified with primers PlXPa and PlXPb to generate a 1.3‐kb fragment using amplification conditions of 94 °C 2 min; 94 °C 15 s; 55 °C 30 s; 70 °C 2 min; repeat 33X; and 72 °C 10 min. DNA products were visualized by agarose gel electrophoresis. Phylogenetic analysis employed Phylogeny.fr 37 using MUSCLE alignment 38 and either maximum likelihood PhyML 3.1^39^ or Bayesian inference Mr. Bayes 3.2.3 40.

### Radiolabel uptake by yeast expressing PlUacP

Time course for the uptake of 0.5 μm [2,8‐^3^H]‐adenine was performed in RG191 cells containing pRH399 or pNS515 and concentrated to a density proportional to OD_600_ = 4. Samplings were harvested at time intervals of 0, 5, 10, 20, 40, 60, and 90 min. Yeast grown for 24 h at 30 °C was concentrated to OD_600_ = 4 and incubated for 0 and 5 min with 0.5 μm **[**8‐^3^H]‐guanine, [2,8‐^3^H]‐adenine, [8‐^3^H]‐xanthine, [5‐^3^H]‐cytosine, [8‐^3^H]‐hypoxanthine, or 1.0 μm [5,6‐^3^H]‐uracil, (Moravek, Brea, CA), in 100 mm citrate buffer (pH 3.5) with 1% glucose. 25 μL aliquots were added to 4 mL of ice‐cold water and filtered through a 0.45 μm Metricel membrane filter (Gelman Sciences, Ann Arbor, MI). Filters were then washed with 8 mL of water, and radioactivity was measured by a Beckman LS 6000IC liquid scintillation counter (Beckman Coulter, Fullerton, CA). Experiments were performed in triplicate. Statistical analysis used an independent paired *t*‐test. Significance was measured at *P* = 0.05 (*), and the error bars represent the standard error of mean.

### Radiolabeled substrate competition study of PlUacP

A substrate competition experiment was performed to further refine the solute binding capacity of PlUacP. Radiolabeled uptake of 1 μm [2, 8‐^3^H]‐adenine was performed in the presence of various heterologous unlabeled competitors including adenine, guanine, cytosine, hypoxanthine, xanthine, uric acid, and uracil. Yeast cells concentrated to OD_600_ ≈ 4 expressing pNS515 were incubated with 1 μm [2, 8‐^3^H]‐adenine in the presence of 1 mm unlabeled heterologous competitors. The samplings were carried out at 5 min for both experiments, and radiolabeled substrate was measured as described above. Experiments were performed in triplicate. Statistical analysis used an independent paired *t*‐test. Significance was measured at *P* = 0.05 (*), and the error bars represent the standard error of mean.

### Transport kinetics and inhibitor studies of PlUacP

The *K*_m_ value of PlUacP for adenine was measured through traditional Michaelis–Menten kinetics experiments. RG191 cells (OD_600_ = 4) containing *PlUacP* were incubated with increasing concentrations of [2,8‐^3^H]‐adenine (0.1, 0.25, 0.5, 1.0, 10.0, 20.0, and 30.0 μm) for 10 min, and the amount of radiolabel taken up by cells was measured as mentioned above. Three independent replicas were used for each substrate concentration. Substrate saturation data were fitted by nonlinear regression. Estimates of *K*_m_ were calculated from the double reciprocal Lineweaver–Burk plot transformation 41. Radiolabeled uptake was performed as previously described at 1 μm [2,8‐^3^H]‐adenine. RG191 cells containing *PlUacP* were incubated with 1 μm [2,8‐^3^H]‐adenine alone or in the presence of 100 μm carbonyl cyanide m‐chlorophenylhydrazone (CCCP) or 1 mm ouabain octahydrate. The reaction was incubated at 30 °C with samples taken at 0 and 5 min, and radioactivity was measured as above. The error bars represent the standard error of the mean of three independent experiments. An independent paired *t*‐test was used to measure statistical significance. Statistical significance was measured at *P* = 0.05 (*).

## Results and Discussion

### *P. larvae* genome contains a locus with similarities to bacterial uric acid and xanthine transporters

**Figure 1:**
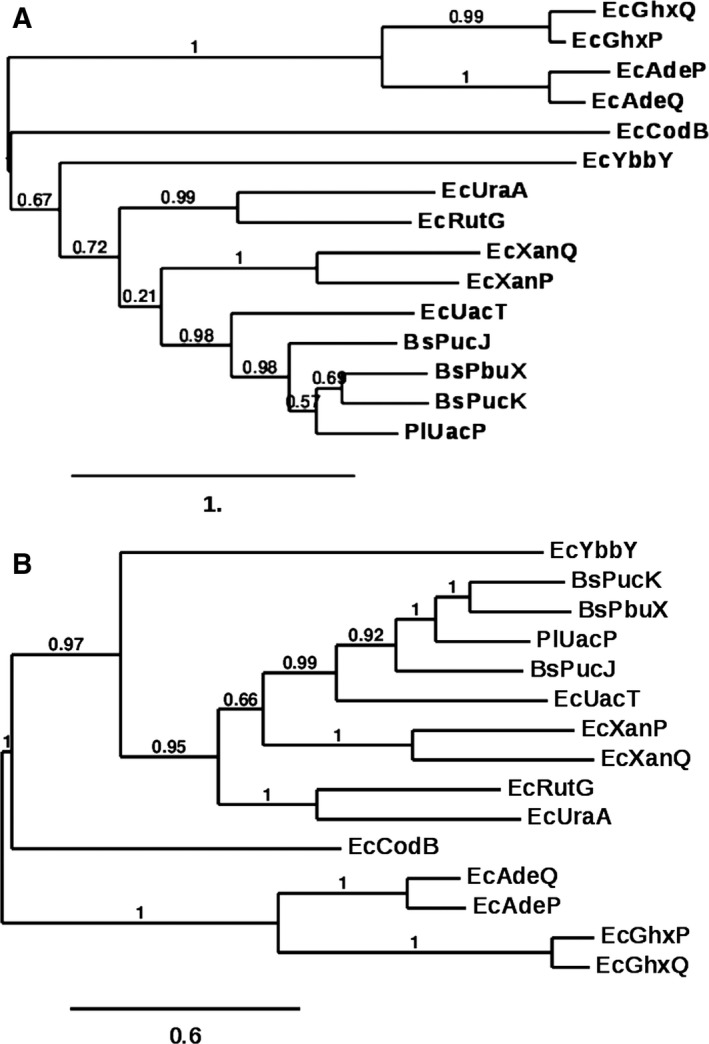
Phylogenetic relationships of PlUacP with select *Escherichia coli* and *Bacillus subtilis* nucleobase transporters. Amino acid sequence accession numbers are given as PlUacP (EFX44190.1); *B. subtilis* PbuX (P42086), PucJ (O32139), PucK (O32140); *E. coli* UraA (BAA16385.1), RutG (P75892.2), XanQ (P67444.2), XanP (P0AGM9.1), UacT (Q46821.1), YbbY (P77328.2), GHXQ (Q46817.2), GHXP (P0AF52.1), AdeP (P31466.2), and AdeQ (P31440.3). Phylogenetic trees were constructed using Phylogeny.fr 37 *,* 2008) using MUSCLE alignment 38 and employing maximum likelihood (A) PhyML 3.1 39 or Bayesian inference (B) Mr. Bayes 3.2.3 40.

**Figure 2:**
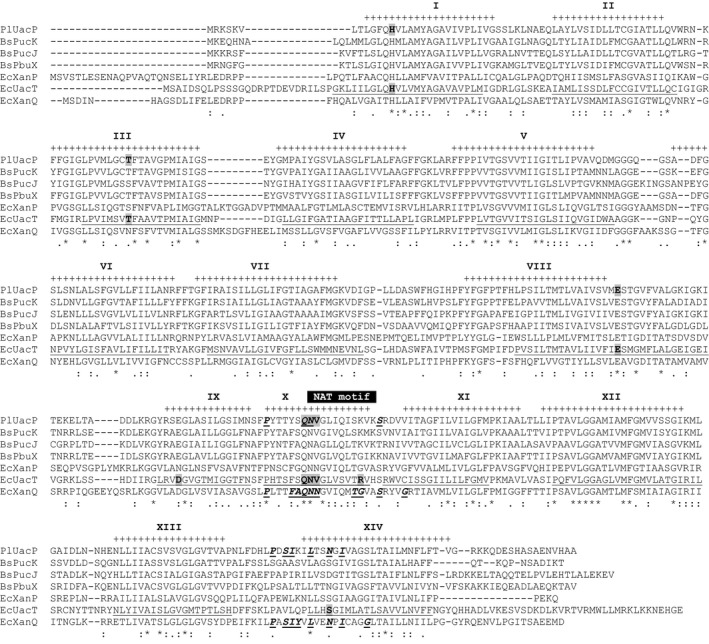
Alignment of PlUacP with select *Escherichia coli* and *Bacillus subtilis* xanthine and uric acid transporters. Alignment of amino acid sequences of *Paenibacillus larvae* UacP with select bacterial xanthine and uric acid transporters as given by ClustalW 55. Amino acid sequences are as designated in Fig 1. The NAT motif conserved among NCS2 proteins is designated by black bar above amino acid sequences 52, 56. Amino acid residues identified by site‐directed mutagenesis in the EcUacT that affect function (bold black text with gray background) are identified 26. Amino acid residues identified by site‐directed mutagenesis in EcXanQ that affect function (bold italic underlined text) are identified 31, 47. Transmembrane spanning domains for PlUacP are indicated by + above amino acid sequence, as predicted by TMHMM v2.0 46 (TM1–TM8 and TM11–TM14) or as identified in EcUacT (TM9 and TM10) 26. Transmembrane spanning domains for EcUacT are identified as underlined text 22.

A search of the *P. larvae* B‐3560 genome through the National Center for Biotechnology Information database with the amino acid sequence of the *E. coli* uric acid transporter UacT (also known as YgfU) using sequence comparison software (tblastn) 42 matched with a presumptive protein (Gb# EFX44190.1) 43. Phylogenetic trees derived from maximum likelihood or Bayesian inference detailing the amino acid sequence comparisons with other characterized nucleobase cation symporter 2 from Gram‐positive *Bacillus subtilis* and Gram‐negative *E. coli* reveal a close association between *P. larvae* protein (Gb# EFX44190.1) and *B. subtilis* and *E. coli* uric acid and xanthine transporters in COG2233 (Fig. 1A,B). Based upon this similarity, the *P. larvae* protein (Gb# EFX44190.1) is now referred to as *P. larvae* uric acid permease‐like protein (PlUacP). PlUacP shares a high level of amino acid similarity with *B. subtilis* uric acid transporters BsPucK (58.9% amino acid identity/87.4% amino acid similarity) and BsPucJ (53.7%/86.2%) 44, xanthine transporter BsPbuX (59.6/90.5%) 45, with lesser, but still substantial, similarity to *E. coli* uric acid transporter EcUacT (38.6%/72.2%) 26, and the xanthine transporters EcXanQ (33.6%/68.9%) and EcXanP (27.1%/62.5%) 25. PlUacP also shares secondary structures, such as fourteen transmembrane spanning (TM) domains, and key signature motifs that are common to uric acid and xanthine transporters as well as other NCS2 (NAT) proteins (Fig. 2). PlUacP contains twelve common overlapping TM domains (TM1–TM8; TM11–TM14) as predicted by secondary structure prediction program TMHMM v2.0 46 when aligned with EcUacT, while the shorter TM domains 9 and 10 present in the crystal structures of EcUraA and AnUapA 24, 30 are not predicted by most secondary structure prediction programs. However, the overall levels of amino acid sequence similarity, common motifs, and transmembrane spanning domain positions are insufficient to assign PlUacP as either a low‐affinity uric acid transporter or a high‐affinity xanthine transporter.

**Figure 3:**
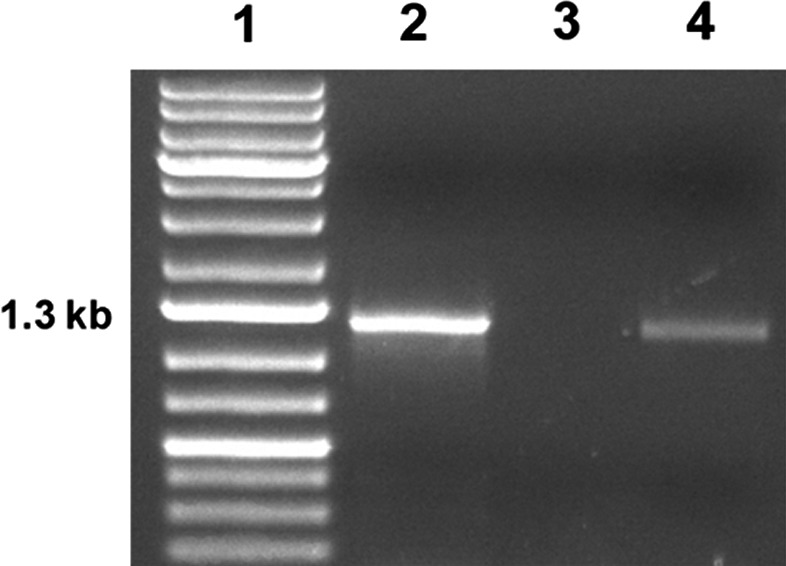
Expression of *PlUacP* by *Paenibacillus larvae*. Expression of *PlUacP* in *P. larvae* by endpoint RT‐PCR. Lane 1, exACTGene 1kb plus DNA ladder (Fisher); lane 2, genomic DNA; lane 3, DNaseI‐treated total RNA without reverse transcription; and lane 4, RT‐PCR of DNaseI‐treated total RNA all amplified with *PlUacP*‐specific primers.

Extensive site‐directed mutagenesis combined with functional analysis of both EcUacT and EcXanQ has identified residues important for transport and solute selectivity. Some of these residues are conserved or present as similar amino acids in PlUacP at the corresponding sites with EcUacT or EcXanQ, while other identified residues are not conserved. Six of nine amino acids previously identified by site‐directed mutagenesis for function in EcUacT are shared in PlUacP (Fig. 2). In EcUacT, aspartic acid (298) essential for function is present as a similar glutamic acid in PlUacP, while serine (426), involved in uric acid affinity in EcUacT, is present as the like amino acid asparagine in PlUacP 26. Similarly, PlUacP shares identity with ten of eighteen amino acids identified as important for function in EcXanQ as determined by site‐directed mutagenesis (Fig. 2) 31, 32, 47. A number of key amino acid residues affecting EcXanQ function in the presumptive solute binding cavity are not conserved in PlUacP: EcXanQ alanine (323) serves to sense xanthine binding and glycine (333) forms part of the purine permeation pathway 47 are, however, present as unlike serine and lysine residues in PlUacP, respectively; phenylalanine (322) and asparagine (326) essential for high‐affinity xanthine binding in EcXanQ 31 are present as unlike tyrosine and valine in PlUacP, respectively; alteration of residue tyrosine (425) in EcXanQ leads to depressed xanthine accumulation 32, but is present as a lysine in PlUacP (Fig. 2). Unfortunately, neither overall amino acid similarities nor the commonality of functionally important residues with other characterized NCS2 proteins are sufficient to assign PlUacP as either a uric acid transporter or a xanthine transporter. An experimental approach to determine function is required to address this question. Endpoint reverse transcription polymerase chain reaction of *P. larvae* RNA from vegetative cells reveals that the *PlUacP* locus is expressed (Fig. 3).

### Heterologous expression of PlUacP in nucleobase transport‐deficient *S. cerevisiae* strains defines solute transport and binding profile

**Figure 4:**
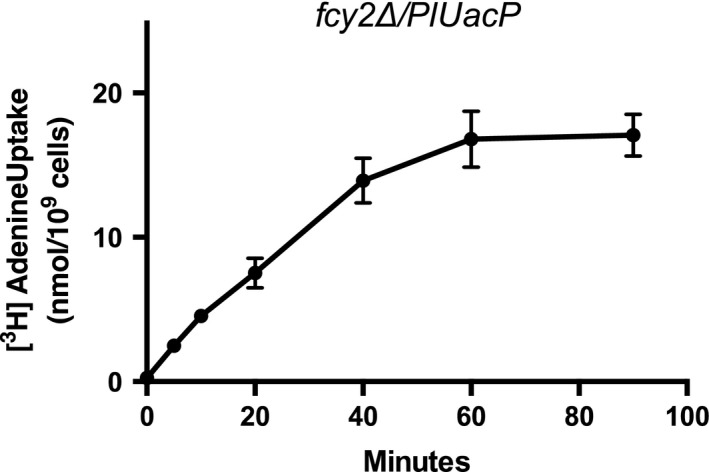
Time‐dependent uptake of [^3^H]‐adenine by yeast containing *PlUacP*. Yeast strain RG191 containing pNS515 (Δ*fcy2*/*PlUacP*) was monitored for the uptake of [2,8‐^3^H]‐adenine over a ninety‐minute time period. Significance was measured at *P* = 0.05 (*). All error bars represent standard error of the mean. Experiments were performed in triplicate.

**Figure 5:**
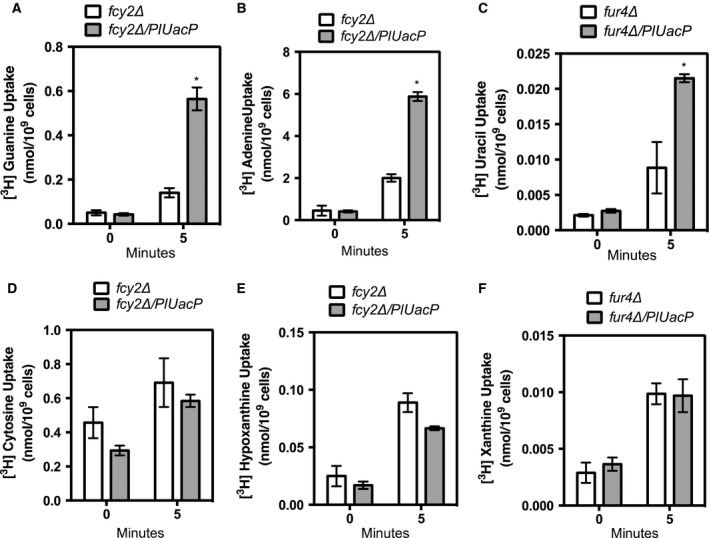
Uptake of radiolabeled nucleobases by yeast containing *PlUacP*. Yeast strain RG191 alone (Δ*fcy2*) or containing pNS515 (Δ*fcy2*/*PlUacP*) was monitored at 0 and 5 min for the uptake of (A) [8‐^3^H]‐guanine, (B) [2,8‐^3^H]‐adenine, (D) [5‐^3^H]‐cytosine, or (E) [8‐^3^H]‐hypoxanthine. Strain NC122‐Sp6 alone or with pNS515 was monitored at 0 and 5 min for the uptake of (C) [5,6‐^3^H]‐uracil or (F) [8‐^3^H]‐xanthine. Statistical analysis used an independent paired *t*‐test. Significance was measured at *P* = 0.05 (*). All error bars represent standard error of the mean. Experiments were performed in triplicate.

Plasmid pNS515 was transformed into *S. cerevisiae* strains RG191 and NC122‐Sp6. Strain RG191 carries a deletion of the *Fcy2* locus that encodes for the guanine–adenine–hypoxanthine transporter and is deficient in transport of these nucleobases. Strain NC122‐Sp6 carries a deletion of the *Fur4* locus that encodes for the uracil transporter and is deficient for transport of uracil. Yeast genomes do not contain loci for xanthine or uric acid transport. These yeast strains and expression plasmids have been successfully employed in heterologous expression experiments monitoring for the uptake of radiolabeled purines and pyrimidines for a variety of plant NCS1 and NCS2 loci for guanine, adenine, cytosine, hypoxanthine, xanthine, and uracil 48, 49, 50, 51, 52. A time course monitoring the uptake of [^3^H]‐adenine for strain RG191 harboring *PlUacP* (RG191/pNS515) displays a saturation after 60 min (Fig. 4). Uptake of [^3^H]‐adenine at 5‐min incubation is within the linear phase and was used as incubation time for subsequent experiments. Figure 5A–C reveals that yeast strains containing pNS515 took up significantly more [^3^H]‐adenine, [^3^H]‐guanine, and [^3^H]‐uracil than strains containing the empty vector. However, no significant difference in uptake was observed for [^3^H]‐cytosine, [^3^H]‐hypoxanthine, or [^3^H]‐xanthine (Fig. 5D–F). PlUacP is an adenine, guanine, and uracil transporter, but not a xanthine transporter. PlUacP stands in contrast to canonical bacterial NCS2 transporters from *E. coli* that tend to be specific for the transport of one or two nucleobases (e.g., *E. coli* uracil transporter 24, xanthine transporters 25, adenine and guanine/hypoxanthine transporters 23).

**Figure 6:**
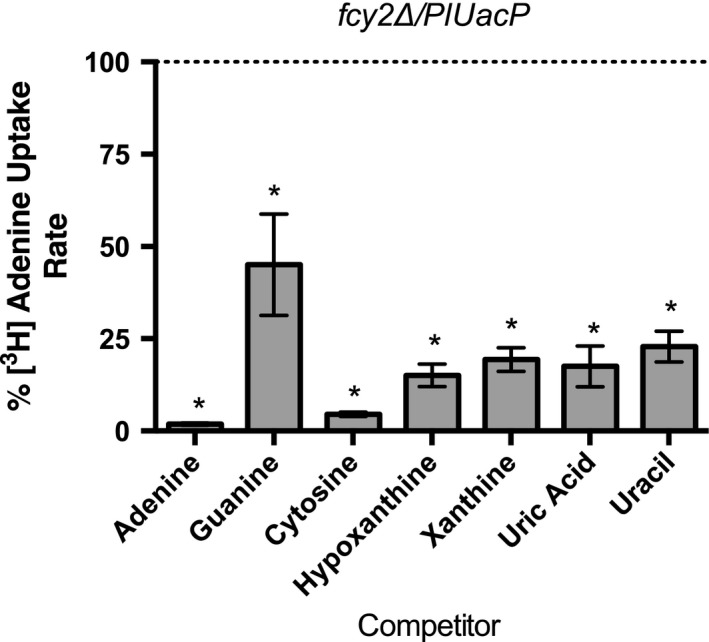
Substrate competition studies of yeast containing *PlUacP*. Yeast strain RG191 containing pNS515 (Δ*fcy2*/*PlUacP*) was monitored for the uptake of [2,8‐^3^H]‐adenine in the presence of excess of unlabeled competitors. Measurements were scored against the uptake of [2,3‐^3^H]‐adenine without competitors as 100% (not shown). Significance was measured at *P* = 0.05 (*). All error bars represent standard error of the mean. Experiments were performed in triplicate.

A series of substrate competition experiments were performed to determine the binding—but not transport—ability of nucleobases on PlUacP. Here, yeast‐harboring *PlUacP* were incubated with radiolabeled adenine alone or with an excess of unlabeled competitors, and the uptake of adenine determined. If competitor molecules bind to the same site in the transporter as does adenine, then adenine transport can be affected—regardless of whether the competitor molecule is actually transported. It is clear that guanine, cytosine, hypoxanthine, xanthine, uric acid, and uracil all compete with adenine and reduce the amount of radiolabel uptake (Fig. 6). The solute transport and binding profile for PlUacP is thus defined as the ability to transport adenine, guanine, and uracil, and to bind cytosine, hypoxanthine, xanthine, and uric acid. This broad nucleobase transport and binding profile differentiates PlUacP from other characterized bacterial NCS2/COG2233 transporters that have both narrow transport and binding profiles (e.g., EcXanQ transports xanthine, but neither transports nor competitively binds adenine, cytosine, hypoxanthine, uric acid, or uracil and displays only weak competitive binding with guanine 25).

The broad solute recognition of PlUacP is reminiscent of some bacterial NCS2 COG2252 and some eukaryotic NCS2 transporters. Recently, it was shown that two AzgA‐like transporters of *P. larvae*, PlAzg1 and PlAzg2, belonging to COG2252, display a wide solute transport profile including adenine, guanine, xanthine, uracil, and cytosine 53. Eukaryotic NCS2 transporters have similarly broad transport profiles including *A. nidulans* AzgA transporting adenine, guanine, and hypoxanthine 19; *Arabidopsis thaliana* Azg1 and Azg2 transporting adenine and guanine; *Zea mays* Azg2–Azg3 transporters transporting adenine, guanine, cytosine, hypoxanthine, and xanthine (Mourad and Schultes, pers. comm.); the *A. thaliana* AtNAT1‐8 and AtNAT12 that transport xanthine, and a combination of adenine, guanine, uracil, and cytosine [18; Mourad and Schultes, pers comm.]. Nonetheless, the solute transport and binding profile for PlUacP is distinct from other NCS2 54. It is clear that despite the close amino acid similarity to other bacterial xanthine or uric acid transporters that have narrow solute transport and binding profiles, PlUacP functions as neither.

### Biochemical and kinetic properties PlUacP differ from the uric acid transporter in *E. coli*

**Figure 7:**
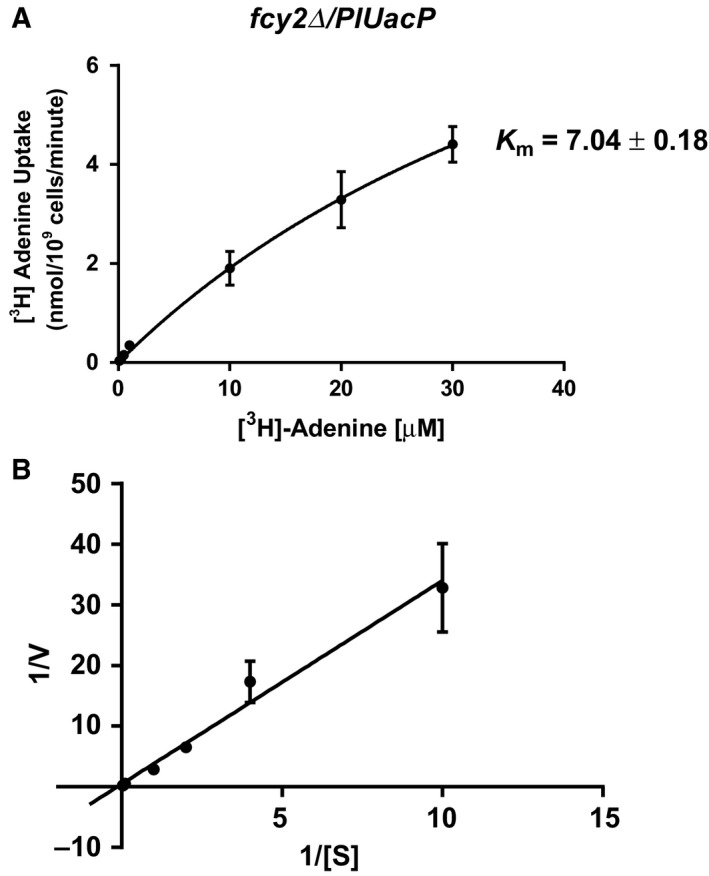
Kinetic values of select nucleobases for PlUacP. Substrate saturation assay in yeast strain RG191 containing pNS515 (Δ*fcy2*/*PlUacP*) with [2,8‐^3^H]‐adenine (A). Double reciprocal Lineweaver–Burk plot (B) for PlUacP was constructed from the substrate saturation curve. All error bars represent standard error of the mean. Experiments were performed in triplicate.

The affinity of PlUacP for adenine was determined using Michaelis–Menten kinetics experiments in which RG191 harboring *PlUacP* was incubated with increasing concentrations of [^3^H]‐adenine and then resulting radiolabel uptake was monitored. Data reveal that PlUacP has a high affinity for adenine with a *K*_m_ 7.04 ± 0.18 μm (Fig. 7A,B), a similar affinity as those determined for other NCS2 adenine transporters EcAdeP (*K*_m_ = 1.0 μm) 23 or AtNAT3 and AtNAT12 (*K*_m_ = 10.12 μm and 1.74 μm, respectively) 18.

Yeast strain RG191 containing *PlUacP* takes up significantly less [^3^H]‐adenine in the presence of the proton uncoupler carbonyl cyanide m‐chlorophenylhydrazone, but not the Na+ gradient disruptor ouabain (Table 1). As with other bacterial, fungal, and plant NCS2, PlUacP appears to be a proton–nucleobase symporter, in contrast to mammalian NCS2 that rely upon a Na+ gradient for function 14. During rapid cell growth in infected honeybee larvae, a rich plethora of compounds released from disrupted cells are available for uptake by the pathogen. Here, it is shown that *P. larvae* has the capacity to import adenine, guanine, and uracil from external sources. This obligate pathogen grows in a nutrient‐rich environment and would benefit from transporters that move a wide range of available nutrients.

**Table 1:** Effects of inhibitors on the function of PlUacP

	%[2,8‐^3^H]‐adenine uptake
RG191/*PlUacP*	100 ± 35.3
RG191/*PlUacP* + *CCCP* *	41.8 ± 1.5
RG191/*PlUacP* + *Ouabain*	114.9 ± 11.3

Significance was measured at *P* = 0.05.

*indicates that the uptake of radio labeled adenine for RG191/PlUacP + CCCP* is significantly different from the uptake of radio labeled adenine for the control RG191/PlUacP.

## Conclusions

Analysis of a nucleobase cation symporter 2 from *P. larvae* reveals a close amino acid sequence similarity to other functionally characterized bacterial xanthine and uric acid transporters. However, functional analysis of PlUacP in a yeast heterologous system reveals it has a broad solute transport and binding profile moving adenine, guanine, and uracil and binding hypoxanthine, xanthine, cytosine, and uric acid, unlike other bacterial xanthine and uric acid transporters. This work reveals that close amino acid sequence similarity by itself cannot be used as a surrogate for assigning function.

## Author contributions

NPS and GSM designed the experiments. AJS and CRA performed the experiments, analyzed the data, and prepared figures. NPS, DWD, and GSM wrote the manuscript.
